# Supplementary material for: Time‑resolved multi-omic analysis of paclitaxel exposure in human iPSC‑derived sensory neurons unveils mechanisms of chemotherapy‑induced peripheral neuropathy
Source: Cell Death Dis. 2026 Feb 10;17(1):211. doi: 10.1038/s41419-026-08445-2 (PMC12921266; doi:10.1038/s41419-026-08445-2)
Supplement: Supplementary file 15 — Dataset 1. [file 41419_2026_8445_MOESM15_ESM.zip › CDD_Open_Data/05_Protein/03_Evidence_Plots_Andranik/GOBP_DENSE_CORE_GRANULE_TRANSPORT.pdf]

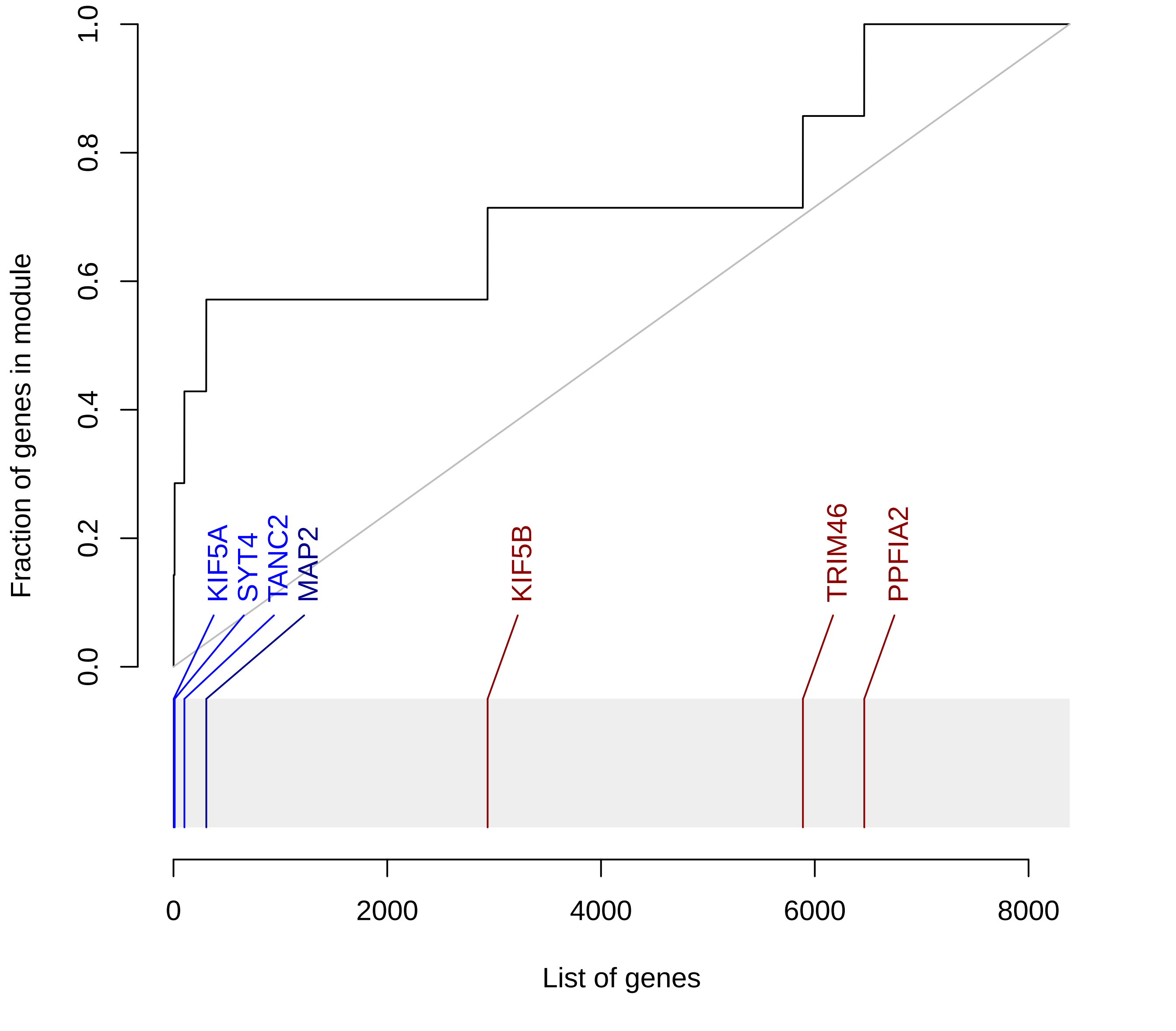

Fraction of genes in module
0.0
0.2
0.4
0.6
0.8
1.0
KIF5A
SYT4
TANC2
MAP2
KIF5B
TRIM46
PPFIA2
0
2000
4000
6000
8000
List of genes
